# Oral health care and mobile ownership in Chinese seniors: CHARLS findings

**DOI:** 10.3389/froh.2025.1632141

**Published:** 2025-09-16

**Authors:** Xiao Hu, Jie Sun, Zhiping Hu

**Affiliations:** 1 Key laboratory of Shaanxi Province for Craniofacial Precision Medicine Research, College of Stomatology, Xi’an Jiaotong University, Xi'an, Shaanxi, China; 2 Chang'an Inkasso, Xi’an, Shaanxi, China

**Keywords:** seniors, dental care utilization, mobile phone ownership, national, region

## Abstract

**Objective:**

Low oral health utilization in elderly individuals is a prominent condition in an aging society. With the increasing coverage of mobile phone ownership, the objective of this study is to empirically examine the relationship between the widespread ownership of mobile phones and the utilization of dental services among the elderly population across the nation.

**Methods:**

We analyzed data from the 2015 wave of the China Health and Retirement Longitudinal Study (CHARLS). The study subjects were elderly individuals aged 60 years or older. The independent variable of oral health care utilization was collected and categorized into “yes” and “no”. To conduct data analysis, we employed the *t*-test for quantitative variables and the chi-square test for qualitative variables. A logistic regression model was constructed to explore the association between oral health care utilization and mobile phone ownership.

**Results:**

The total sample size in our study was 9,861. Among elderly individuals, 18.9% reported utilizing dental services within the preceding year. The sample's demographic profile revealed that 39.7% of participants were aged 75 years or older, and 49.7% were female. Age, region, satisfaction with local healthcare services, and mobile phone ownership were significantly associated with dental service utilization. Among elderly individuals, regional variations significantly influenced the prevalence of dental care utilization among elderly individuals with (OR = 1.30, 95% CI: 0.06–0.24) and without mobile phones (OR = 1.29, 95% CI: 0.08–0.25), as assessed by oral health utilization. Furthermore, those satisfied with local health services exhibited a higher likelihood of service utilization, irrespective of mobile phone ownership status (OR = 0.86, 95% CI: −0.14 to 0.03) vs. (OR = 0.90, 95% CI: −0.11 to 0.04).

**Conclusion:**

The utilization rate of oral health utilization was low in older Chinese individuals. With respect to mobile phone ownership, we found that region significantly increased the likelihood of oral health utilization in the adjusted model. Our results may be useful for the development of policies aimed at achieving greater oral health utilization in older adults with mobile phones, especially those who are not living in urban areas.

## Introduction

Elderly individuals aged 60 years and older have urgent dental health needs. Nearly 19% of these patients no longer have any natural teeth (1), and teeth become darker with age (2). A systematic review revealed that the deterioration of the oral health status of elderly individuals; a decline in oral motor skills; chewing, swallowing and saliva disorders; and oral pain are associated with adverse health outcomes (3). Higher-quality studies of interventions in older people's oral health are needed.

With the aging of the population, the factors impacting the utilization of dental health services by aged mouths are becoming increasingly prominent. Previous studies have shown that socioeconomics, lifestyle and the environment are vital aspects of access to oral health care (4). A cross-sectional study by Xu et al. (5) revealed a strong association between perceived oral health status, dental pain and oral health service utilization among preschool children, whereas the relationships among adults and older adults (>60 years) are more mixed (6, 7). Interpretive research has revealed that mobile phones have the potential to be an innovative way to promote prenatal oral care use (8). Lectures of good oral health for this cohort were the preferred way to improve the oral health of older people (9). Additionally, systematic reviews and randomized control trials have demonstrated that smartphone applications are underutilized in dentistry (10).

Currently, as the use of mobile phone increases and its new model in dentist (11, 12), mobile phone ownership is a largely untapped resource in dentistry, especially for the elderly (13, 14). Studies indicate that mobile health (mHealth) applications and tele-dental services facilitate easier scheduling of appointments and provide remote consultation options, which are particularly beneficial for elderly individuals with limited mobility or those residing in remote areas (15–17). Furthermore, regular push notifications about oral health education improve adherence to preventive measures (18). However, the digital divide, including lower digital literacy and technical challenges, remains a barrier for some elderly individuals, highlighting the need for user-friendly interfaces and targeted training programs (19). Overall, mobile phone ownership can significantly improve oral health outcomes among the elderly, provided that appropriate support and interventions are in place to address existing challenges.

The aim of our study was to identify the association between oral health utilization and this tool among the elderly at the national level.

## Methods

### Data source and sample selection

Deidentified data from the third wave in 2015 of the China Health and Retirement Longitudinal Study (CHARLS), obtained from the National Development Research Institute of Peking University and coexecuted by the Chinese Social Science Survey Center of Peking University and the Peking University Youth League Committee, were analyzed to assess oral health utilization. To be included in our study, respondents needed to be aged 60 years and older and have complete data on age, sex, dental health utilization and mobile phone ownership. In the end, a final sample of 9,861 was included in our study. The key design of the CHARLS project has been discussed elsewhere (20, 21) and on the official website (https://charls.pku.edu.cn/). The Ethics Committee of Peking University ratified the CHARLS study, and all participants signed informed consent forms.

### Variables

The explained variable in this study was oral health utilization, which was based on the following question: “In the past year, have you seen a dentist for dental care, including dentures?”, respondents choose from yes or no. The explanatory variable was mobile phone ownership. The question in the questionnaire was “Do you and your spouse own the following assets?” There were 18 options for this question, and the mobile phone was one of the assets; the answer was yes or no. As covariate variables, demographic characteristics and local health service satisfaction, including age, sex, marriage and household registration type, were added. We divided the continuous age data into three categories: 60–70 years (including 60), 70–75 years (including 70) and 75 years and older. The survey included seven options regarding marital status: “Married with spouse present, Married but temporarily apart from spouse due to circumstances like work, separated, divorced, widowed, never married, and cohabited.” We grouped these statuses into three categories: “be Marriage” (which includes “Married with spouse present” and “Married but temporarily apart from spouse for reasons such as work”), “Widowed,” and “Others” (which encompasses all other marital statuses). Additionally, the question provided seven options regarding participants living address: Main city zone, a combination of urban and rural areas, the town center, ZhenXiang area, special area, Township central, and Village. We classified these into three categories: urban (Main city zone), rural (Village), and others (combination of urban and rural areas, the town center, ZhenXiang area, special area, Township central). “Are you satisfied with the quality, cost, and convenience of local health care services?” This was the other type of confounding variable question, with response options ranging from 1 to 5, where 1 indicates “very satisfied” and 5 signifies “very dissatisfied.”

### Statistical analyses

Percentages were calculated for categorical data, and the chi-square test was used to assess differences. The analysis is divided into two groups: the elderly with mobile phones and the elderly without mobile phones. The results of logistic regression are presented as regression coefficients (B), 95% confidence intervals (95% CI), and *p*-values for various factors.

The factors included in our study were all examined in the regression models, with variables found to be associated with oral health utilization at *P* < .05 retained in the models. Model 1 was adjusted for age, sex, household registration type and marital status. Model 2 included Model 1 demographic factors plus local health service satisfaction. All analyses were performed via SPSS Statistics, version 27.0 (IBM Corp).

## Results

### Descriptive statistics

The study sample characteristics are summarized in Table 1. According to the study purpose and missing data, our total sample size was 9,861, and the proportion of patients using dental services over the past year was 1,862 (18.9%). The number of individuals above 75 years of age in the study population was 3,915 (39.7%), and 4,904 (49.7%) were females. Table 1 also presents the baseline characteristics of 7,087 (71.9%) participants who were registered under a rural household classification and 7,807 (79.2%) who were married in the cross-sectional study. There were 5,255 (53.3%) mobile phone users and 3,623 (36.7%) participants were neutral with respect to local health services. A comparison of unadjusted rates revealed that five indicators were significantly associated with OHC utilization (see Table 1).

**Table 1:** Characteristics of the study population in 2015.

Variables		*N*	%
See a dentist over the past year	Yes	1,862	18.9
	No	7,999	81.1
Age*	60–70 years old(including 60)	3,188	32.2
	70–75 years old(including 70)	2,758	28.0
	75 years and older	3,915	39.7
Sex*	Male	4,957	50.3
	Female	4,904	49.7
Household registration type*	Urban	1,485	15.1
	Rural	7,087	71.9
	Others	1,289	13.1
Marital status	Be marriage	7,807	79.2
	Widowed	1,517	15.4
	Others	537	5.4
Mobile phone ownership*	Yes	5,255	53.3
	No	4,606	46.7
Satisfied with the local health care services*	Very satisfied	1,701	17.2
	Somewhat satisfied	2,570	26.1
	Neutral	3,623	36.7
	Somewhat dissatisfied	1,240	12.6
	Very dissatisfied	727	7.4
Total		9,861	100

* *P* < 0.05 indicates a statistically significant difference.

An analysis was carried out on the services utilized by participants who possessed mobile phones in comparison to those who did not. This examination revealed three factors that exhibited statistical significance among older adults with mobile phones (refer to Table 2), while four factors indicated statistical significance among older adults without mobile phones (refer to Table 3). Variables such as age, type of household registration, and contentment with local health care services are important in influencing oral health utilization in both groups. It is clear that elderly individuals with mobile phones report a slightly higher percentage of “Yes” responses regarding oral health utilization (20.4%) compared to those without mobile phones (17.1%). Among the elderly without mobile phones, the marital status of “being married” (17.8%) was associated with significantly greater oral health utilization.

**Table 2:** Oral health utilization among the elderly with mobile phones.

Variables		Yes *N*(%)	No *N* (%)	Total *N*(%)
Sex	Male	580 (19.5)	2,393 (80.5)	2,973 (56.6)
	Female	493 (21.6)	1,789 (78.4)	2,282 (43.4)
Age*	60–70 years old(including 60)	350 (20.3)	1,377 (79.7)	1,727 (32.9)
	70–75 years old(including 70)	343 (22.6)	1,175 (77.4)	1,518 (28.9)
	75 years and older	380 (18.9)	1,630 (81.1)	2,010 (38.2)
Household registration type*	Urban	244 (29.2)	593 (70.8)	837 (15.9)
	Rural	670 (18.2)	3,016 (81.8)	3,686 (70.1)
	Others	159 (21.7)	573 (78.3)	732 (13.9)
Marital status	Be marriage	787 (20.9)	2,983 (79.1)	3,770 (71.7)
	Widowed	221 (19.5)	914 (80.5)	1,135 (21.6)
	Others	65 (18.6)	285 (81.4)	350 (6.7)
Satisfied with the local health care services*	Very satisfied	135 (12.6)	681 (16.3)	816 (15.5)
	Somewhat satisfied	247 (23.0)	1,090 (26.1)	1,337 (25.4)
	Neutral	406 (37.8)	1,526 (36.5)	1,932 (36.8)
	Somewhat dissatisfied	187 (17.4)	569 (13.6)	756 (14.4)
	Very dissatisfied	98 (9.1)	316 (7.6)	414 (7.9)
	Total	1,073 (20.4)	4,182 (79.6)	5,255 (100)

* *P* < 0.05 indicates a statistically significant difference.

**Table 3:** Oral health utilization among the elderly without mobile phones.

Variables		Yes *N*(%)	No *N* (%)	Total *N*(%)
Sex	Male	316 (15.9)	1,668 (84.1)	1,984 (43.1)
	Female	473 (18.0)	2,149 (82.0)	2,622 (56.9)
Age*	60–70 years old(including 60)	253 (17.3)	1,208 (82.7)	1,461 (31.7)
	70–75 years old(including 70)	258 (20.8)	982 (79.2)	1,240 (26.9)
	75 years and older	278 (14.6)	1,627 (85.4)	1,905 (41.4)
Household registration type*	Urban	156 (24.1)	492 (75.9)	648 (14.1)
	Rural	536 (15.8)	2,856 (84.2)	3,401 (73.8)
	Others	97 (17.4)	469 (82.6)	557 (12.1)
Marital status*	Be marriage	717 (17.8)	3,320 (82.2)	4,037 (87.6)
	Widowed	42 (11.9)	340 (88.1)	382 (4.1)
	Others	30 (16.0)	157 (84.0)	187 (8.3)
Satisfied with the local health care services*	Very satisfied	137 (15.5)	748 (84.5)	885 (19.2)
	Somewhat satisfied	200 (16.2)	1,033 (83.8)	1,233 (26.8)
	Neutral	284 (16.8)	1,407 (83.2)	1,691 (36.7)
	Somewhat dissatisfied	94 (19.4)	390 (80.6)	484 (10.5)
	Very dissatisfied	74 (23.6)	239 (76.4)	313 (6.8)
	Total	789 (17.1)	3,817 (82.9)	4,606 (100)

* *P* < 0.05 indicates a statistically significant difference.

### Main results of logistic regression analyses

Logistic regression analyses stratified by socioeconomic factors were performed separately among the elderly individuals with and without mobile phones to explore factors associated with oral health utilization. The differences in the utilization of dental health services between the two groups were inconsistent (see Table 4).

**Table 4:** Logistic regression of oral health utilization among the elderly by mobile phone ownership group.

Variables	Model 1				Model 2			
	OR	95%CI		*P* value	OR	95%CI		*P* value
		Lower	Upper			Lower	Upper	
The elderly with mobile phone								
Sex	.87	.76	1.00	.05	.86	−.16	−.07	.03
Age	1.04	.96	1.13	.31	1.10	−.03	.04	.43
Household registration type	1.28	1.13	1.45	<.001	1.30	.06	.24	<.001
Marital status	1.10	.98	1.24	.11	1.22	−.06	.09	.13
Satisfied with the local health care services					.86	−.14	−.03	<.001
The elderly without mobile phone								
Sex	.86	.76	1.00	.06	.84	−.17	−.08	.03
Age	1.10	1.01	1.21	.03	1.09	−.09	1.05	.06
Household registration type	1.30	1.12	1.51	<.001	1.29	.08	.25	<.001
Marital status	1.22	1.02	1.46	.03	1.22	.09	.20	.03
Satisfied with the local health care services					.90	−.11	−.04	.002

### The elderly with mobile phones

Among elderly individuals who own mobile phones, the logistic regression analysis shows clear trends in the use of oral health services. In Model 1, being female is linked to slightly decreased odds of using oral health services (OR = 0.87, 95% CI: 0.76–1.00), while age indicates a minor positive correlation (OR = 1.04, 95% CI: 0.96–1.13). The type of household registration significantly impacts utilization positively (OR = 1.28, 95% CI: 1.13–1.45), and marital status suggests a mild positive correlation (OR = 1.10, 95% CI: 0.98–1.24). In Model 2, the negative correlation for females becomes more pronounced (OR = 0.86, 95% CI: 0.76–1.00), whereas age presents a substantial positive association (OR = 1.10, 95% CI: 1.00–1.20). The significance of household registration type remains positively linked (OR = 1.30, 95% CI: 1.14–1.48), and marital status also indicates a significant positive connection (OR = 1.22, 95% CI: 1.06–1.40). Furthermore, satisfaction with local healthcare services is inversely related to the use of oral health services (OR = 0.86, 95% CI: 0.78–0.95).

### The elderly without mobile phones

For the elderly without mobile phones, the logistic regression analysis also highlights specific factors influencing oral health service utilization. In Model 1, being female is associated with a marginally significant negative impact (OR = 0.86, 95% CI: 0.76–1.00), and age shows a significant positive association (OR = 1.10, 95% CI: 1.01–1.21). Household registration type has a significant positive impact (OR = 1.30, 95% CI: 1.12–1.51), and marital status shows a significant positive association (OR = 1.22, 95% CI: 1.02–1.46). In Model 2, the negative association for females becomes more significant (OR = 0.84, 95% CI: 0.75–0.95), and age shows a marginally significant positive association (OR = 1.09, 95% CI: 1.00–1.19). Household registration type remains significantly positive (OR = 1.29, 95% CI: 1.08–1.54), and marital status also shows a significant positive association (OR = 1.22, 95% CI: 1.02–1.46). Furthermore, satisfaction with local health care services is negatively associated with oral health service utilization (OR = 0.90, 95% CI: 0.83–0.98).

## Discussion

This study has two main objectives, and the obtained results are worthy of in-depth exploration. First, from a national perspective, we conducted research on the relative utilization of oral health services among the elderly population in China, aiming to comprehensively understand the current actual situation.

There are several recognized relationships among health service utilization, age groups, and oral health. Our data are consistent with these well-known relationships. For example, the utilization rate of the 75 years old and above age group is the lowest. This may be because as people age, their physical functions gradually decline, and they become less mobile (22, 23). Visiting a dental clinic can be difficult for them, such as facing transportation inconvenience or having trouble independently completing complex medical treatment procedures. These factors increase the difficulty of accessing oral health services, thus resulting in a relatively low utilization rate. This oral health disparity caused by accessibility issues is consistent with the findings of previous related studies on the oral health of elderly people. Previous studies have emphasized that oral health should be regarded as a key indicator of healthy aging, which has received increasing attention in the field of public health (4).

In fact, it is quite challenging to achieve good oral health by increasing the utilization rate of dental services, especially for elderly individuals. Our descriptive statistical results show that the utilization rate of dental services among elderly individuals in China is rather low, which is consistent with the conclusions of earlier studies that elderly individuals who visit dentists usually have relatively good health conditions (24). This means that those elderly people with more serious oral problems and limited mobility may be unable to obtain the necessary oral medical services in a timely manner due to various obstacles, making it difficult to effectively improve their overall oral health level.

Second, this study aims to empirically test the hypothesis that “owning a mobile phone can promote the utilization of oral health services”. Although we need to be cautious when interpreting the research results before conducting more in-depth studies, considering factors related to patients, it can be reasonably inferred that the ownership of mobile phones is one of the factors influencing the utilization of dental services. In today's society, mobile phones are not just simple communication tools; they also have the functions of accessing rich information and interacting (25, 26). Elderly individuals who own mobile phones can easily obtain oral health knowledge through their phones, such as the correct way of brushing their teeth and preventive measures for oral diseases (27), thereby enhancing their oral health awareness and increasing their likelihood of actively seeking oral health services. Moreover, mobile phones also help elderly people access health services (28), simplify the medical treatment process, and reduce the difficulty of seeking medical treatment. For example, some dental medical institutions have launched online appointment platforms, and elderly individuals can easily make appointments for suitable visit times through their mobile phones, reducing the time and energy spent waiting in line (29, 30).Notably, this hypothesis is highly consistent with the existing research results. These studies clearly indicate that the lack of mobile phones can be a major obstacle to the successful implementation of mHealth interventions (31). In the field of oral health, interventions can use mobile phone applications to push personalized oral health advice and remind patients to return for follow-up consultations in time (13, 32). Elderly individuals without mobile phones cannot enjoy these convenient mHealth services and are at a disadvantage in obtaining oral health information and services. This further highlights the potential value of mobile phones in enhancing the utilization of oral health services among elderly individuals.

In addition, this study revealed that regional factors play an important regulatory role in the relationship between mobile phone ownership and the utilization of oral health services. Different regions have different economic development levels, distributions of medical resources, and cultural concepts (33). These differences can affect the ability of elderly people to use mobile phones and their acceptance of them, thus influencing the ability of mobile phones to promote the utilization of oral health services. In regions with developed economies, rich medical resources, and high-level informatization, the elderly may be more likely to access and proficiently use mobile phones to obtain oral health services (34, 35). However, in relatively underdeveloped regions with scarce medical resources, even if the elderly own mobile phones, owing to the limited accessibility of local oral medical services, the role of mobile phones in promoting the utilization of oral health services will be greatly restricted. Community-based programs pairing mobile education with local dental outreach may maximize impact, especially in underserved regions. The coexistence of high mobile phone ownership yet low service use in rural elderly may reflect accessibility barriers (e.g., transportation, clinic availability) or digital literacy gaps. Future interventions could leverage basic phone functions (e.g., voice reminders) rather than complex apps.

### Limitations

However, this study also has certain limitations. First, the data are from a 2015 survey. Over time, the social environment, lifestyle, popularity and functions of mobile phones have changed. The research results may not fully reflect the current actual situation. This study exclusively examined the association between mobile phone ownership among elderly residents in urban, rural, and other predefined regions and their utilization of oral health services, without addressing refined residential classifications (e.g., a combination of urban and rural areas), frequency of device engagement. Future research can conduct further follow-up surveys to obtain more timely data and, at the same time, analyze the relationship between mobile phone usage behavior and the utilization of oral health services in detail, providing a more solid basis for formulating more effective policies and measures to promote the oral health of elderly individuals.

## Conclusion

For future policy making, it is worth the effort to rely on mobile phones to explore appropriate health promotions to increase oral health service use and decrease the degree of underutilization of dental services among elderly individuals.

## Data Availability

Publicly available datasets were analyzed in this study. This data can be found here: https://charls.pku.edu.cn/.
